# A new approach to improve the electrochemical performance of ZnMn_2_O_4_ through a charge compensation mechanism using the substitution of Al^3+^ for Zn^2+^

**DOI:** 10.1039/c8ra00310f

**Published:** 2018-02-15

**Authors:** Xianyu Zhu, Jingbin Quan, Jichun Huang, Zheng Ma, Yixin Chen, Decheng Zhu, Chongxing Ji, Decheng Li

**Affiliations:** College of Physics, Optoelectronics and Energy, Soochow University Soochow People's Republic of China lidecheng@suda.edu.cn

## Abstract

ZnMn_2_O_4_ and Zn_1−*x*_Al_*x*_Mn_2_O_4_ were synthesized by a spray drying process followed by an annealing treatment. Their structural and electrochemical characteristics were investigated by SEM, XRD, XPS, charge–discharge tests and EIS. XPS data indicate that the substitution of Al^3+^ for Zn^2+^ causes manganese to be in a mixed valence state by a charge compensation mechanism. Moreover, the presence of this charge compensation significantly improves the electrochemical performance of Zn_1−*x*_Al_*x*_Mn_2_O_4_, such as increasing the initial coulombic efficiency, stabilizing the cycleability as well as improving the rate capability. The sample with 2% Al doping shows the best performance, with a first cycle coulombic efficiency of 69.6% and a reversible capacity of 597.7 mA h g^−1^ after 100 cycles. Even at the high current density of 1600 mA g^−1^, it still retained a capacity of 558 mA h g^−1^.

## Abstract 2

This work reports the nonequivalent substitution of ZnMn_2_O_4_. This is a new approach to improve the electrochemical performance of ZnMn_2_O_4_ through a charge compensation mechanism using the substitution of Al^3+^ for Zn^2+^.

## Graphical abstract

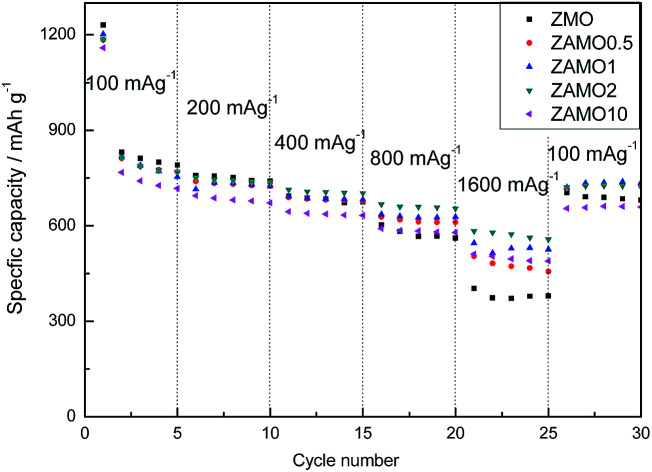

## Introduction

Recently, rechargeable lithium ion batteries (LIBs), as energy storage devices, have been wildly applied in hybrid electric vehicles (HEVs), pure electric vehicles (EVs) and plug-in hybrid vehicles (PHEVs). However, in these cases, consumers want the vehicles to have a long cruising mileage and good safety, which requires LIBs with enhanced performance in terms of their energy density and safety.^1–4^ Most commercial LIBs still use graphite as the anode material due to its low cost, stable capacity, and long cycle life.^5^ However, the structure of graphite means that its theoretical capacity is only 372 mA h g^−1^ and the practical capacity is near to 360 mA h g^−1^. Thus, the demand for next-generation LIBs with higher capacity has stimulated efforts to develop new materials^6,7^ such as graphene, carbon nanotubes, silicon, tin oxide, *etc.*

These new materials can usually be classified into three groups in terms of their electrochemical mechanism.^8^ The first is the intercalation/de-intercalation type, including many carbonaceous materials like hard carbons,^9–11^ carbon nanotubes^12–15^ and graphene,^16^ and some titanium oxides, such as TiO_2_ (ref. 17) and LiTi_4_O_5_.^17^ The second type is alloy/de-alloy mechanism. This type materials include such as silicon,^18^ germanium,^19,20^ tin,^21^ antimony,^22^ tin oxide^23^ and SiO.^24^ The third type is usually named as conversion mechanism mainly referring to some transition metal oxides such as manganese oxide,^25–27^ cobalt oxide^28^ and iron oxide.^29^ These materials have the advantages of high capacity, high energy, low cost and environmentally compatibility but also exist disadvantages like low coulombic efficiency, unstable SEI formation, large potential hysteresis and poor cycle life.

ZnMn_2_O_4_ belongs to the third type mechanism and not only has the same advantages, but also has an appropriated working potential above lithium, which could restrain the formation of lithium dendrites. However, ZnMn_2_O_4_ has two obvious drawbacks. One is poor electrical conductivity. The other is instability during the charge/discharge process. ZnMn_2_O_4_ can't be commercialized unless these two issues are solved.

So far, the investigations on the structural and electrochemical performances of the ZnMn_2_O_4_ are inadequate and rather few works were reported. Deng *et al.* synthesized agglomerated pure spinel ZnMn_2_O_4_*via* calcination of an agglomerated Zn–Mn citrate complex precursor maintained a specific capacity of 650 mA h g^−1^ over 200 cycles.^30^ Courtel *et al.* synthesized nanoparticles ZnMn_2_O_4_ showed a capacity of 690 mA h g^−1^ after 90 cycles by using co-precipitation method.^31^ Feng *et al.* successfully synthesized ZnMn_2_O_4_ with the particle size about 50 nm retained a capacity of 745 mA h g^−1^ after 160 cycles through a rheological phase method.^32^

The groups mentioned above usually focused on the preparation of nano-size ZnMn_2_O_4_.^33^ It is well known that nano-size materials have many merits such as high surface area, short diffusion distance. However, the nano size materials usually need more binder as well as more conductive carbon in the formation of the electrode. The smaller the particle size is, the more binder amount needs.

It is well known that foreign metal ions doping can improve the electrochemical performances of the cathode materials. Generally, there are two kinds of substitution in terms of the charge balance.^34^ One is the equivalent and the other is the nonequivalent substitution. The equivalent-substitute ZnMn_2_O_4_, such as Cd-doped ZnMn_2_O_4_,^35^ has been studied already. However, investigations of the nonequivalent-substituted ZnMn_2_O_4_ are rather limited.

In our work, we want to improve the electrochemical performances through the nonequivalent substitution. ZnMn_2_O_4_ and Zn_1−*x*_Al_*x*_Mn_2_O_4_ were synthesized by spray drying process following with annealing treatment. We also investigated the influence of aluminum doping on the structural and electrochemical performances of ZnMn_2_O_4_.

## Experimental

ZnMn_2_O_4_ and Zn_1−*x*_Al_*x*_Mn_2_O_4_ (*x* = 0.5%, 1%, 2%, 10%, mark as ZAMO0.5, ZAMO1, ZAMO2, ZAMO10, respectively) were prepared by a typical spray drying method using a citrate ligand to complex the metal ions. For this purpose, Zn(Ac)_2_·H_2_O (0.05, 0.04975, 0.0495, 0.049, 0.045 mol), Al(Ac)_3_·9H_2_O (0, 0.00025, 0.0005, 0.001, 0.005 mol), Mn(Ac)_2_·4H_2_O (0.1 mol) and citric acid (0.05 mol) were dissolved in 125 ml of deionized water with vigorous agitation. The mixed solution was spray-dried as the outlet temperature was set to 185 °C. Then the obtained white acetate powder was pre-sintered at 300 °C in air for 3 hours to decompose the organic constituents. After grinding into powder, the precursor was sintered at 600 °C in air for 20 hours to obtain target products.

## Characterization

The phase composition and structure of the sample were performed using an X-ray diffractometer equipped with Cu Kα radiation (XRD, Rint 1000, Rigaku, Japan) over the 2*θ* range of 10–90°. The morphologies and sizes of the samples were directly examined by scan electron microscopy (SEM). The electronic states of Mn in the samples were determined X-ray photoelectron spectroscopy (XPS, ESCALAB 250Xi).

CR2032 coin-tape cell was used to investigate the electrochemical performances of the synthesized materials. The active material was mixed in slurry containing 10 wt% of Super P as a conductive agent and 10 wt% NaCMC as a binder. The homogeneous slurries were cast onto copper foils to obtain electrode laminate which were dried at 110 °C in the vacuum drying oven all over the night. The working cell was assembled in a glove box filled with dry argon. A lithium disk (*Φ* 15 × 1 mm) was used as a negative electrode (counter electrode and reference electrode). A Celgard 2400 porous polypropylene film was served as a separator. The electrolyte was 1 M LiPF_6_ dissolved in a compound of diethyl carbonate/ethylene carbonate (DEC/EC, 1 : 1 by volume). The galvanostatic charge–discharge cycling and cyclic voltammetry were tested at room temperature (RT ∼ 25 °C) by means of a computer-controlled battery evaluation system (LAND CT 2001, Wuhan, China). And the electrochemical impedance spectroscopy (EIS) measurements were performed *via* PARSTAT 2273 electrochemical workstation system (Princeton Applied Research, AMETEK, America) over frequency range of 100 kHz to 10 mHz with amplitude of 10 mV.

## Results and discussion

**Fig. 1:**
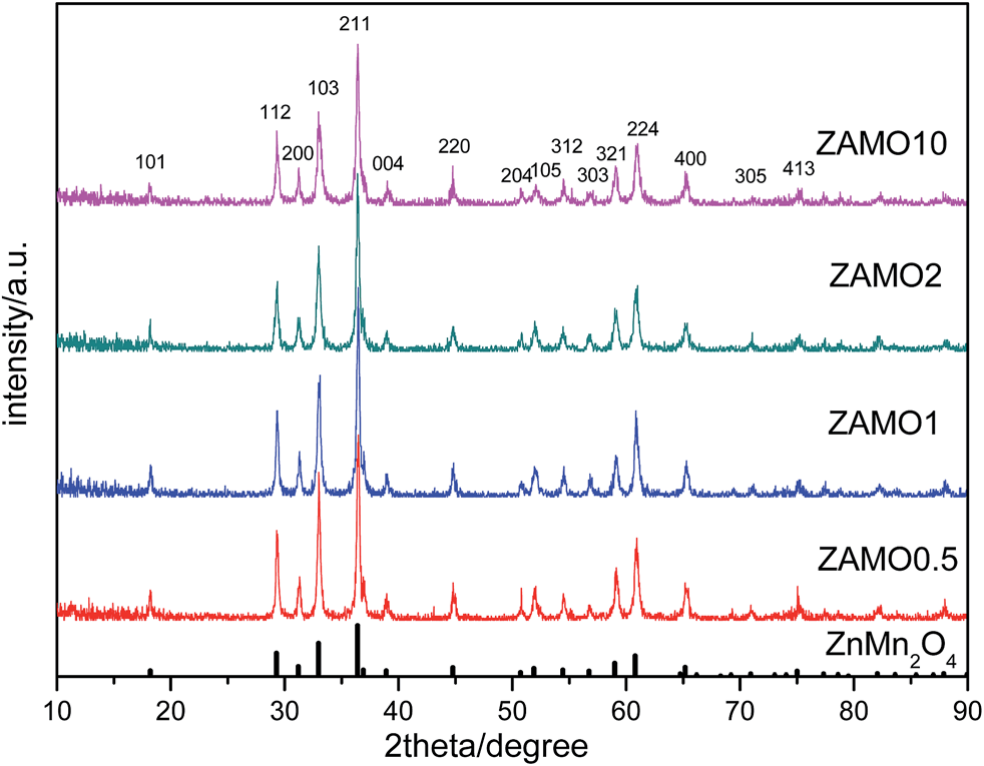
XRD patterns of Zn_1−*x*_Al_*x*_Mn_2_O_4_ samples.

**Fig. 2:**
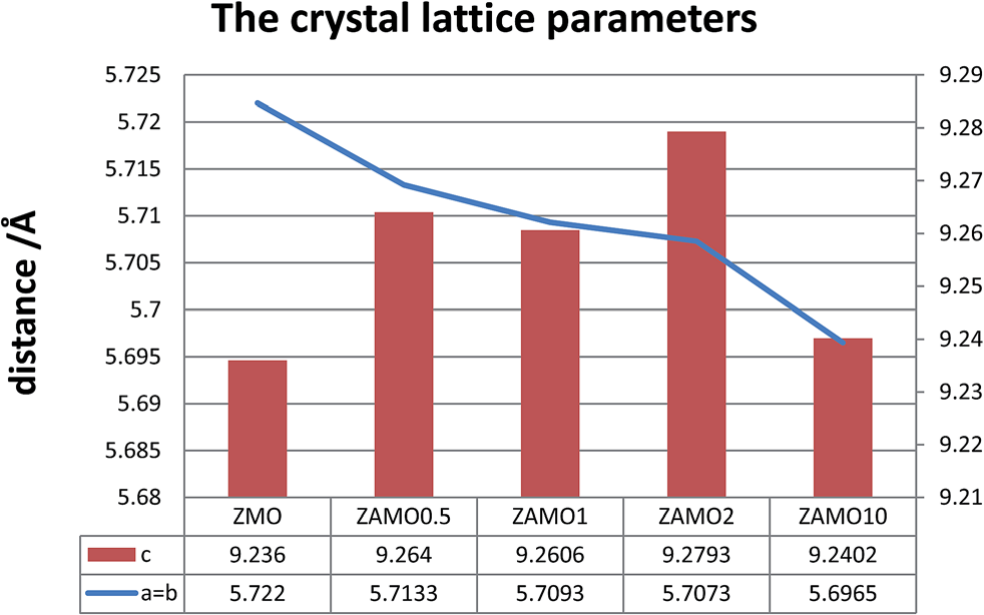
The calculated lattice parameters of ZnMn_2_O_4_ and Zn_1−*x*_Al_*x*_Mn_2_O_4_.

XRD patterns of the Zn_1−*x*_Al_*x*_Mn_2_O_4_ materials are shown in Fig. 1. All diffraction peaks can be indexed well to tetragonal ZnMn_2_O_4_ (JCPDS card no. 24-1133; space group: *I*4_1_/*amd*). No additional peaks are observed, indicating that the samples are pure. The lattice parameters calculated by JADE are shown in Fig. 2. The lattice parameters of the Zn_1−*x*_Al_*x*_Mn_2_O_4_*a*/*b* are gradually decreased from 5.722 Å to 5.6965 Å when *x* is increased from 0 to 10%. In the case of lattice parameter *c*, it is initially increased from 9.236 Å to 9.2793 Å when *x* is increased from 0 to 2%, then decreased to 5.6965 Å when *x* is increased 10%. This variation of lattice parameters should be ascribed to the substitution of Al^3+^ to Zn^2+^ since the radius of the Al^3+^ is smaller than that of the Zn^2+^ (Al^3+^: 0.535 Å, Zn^2+^: 0.74 Å). These results also indicate that Al^3+^ has been successfully introduced into the ZnMn_2_O_4_ lattice.

**Fig. 3:**
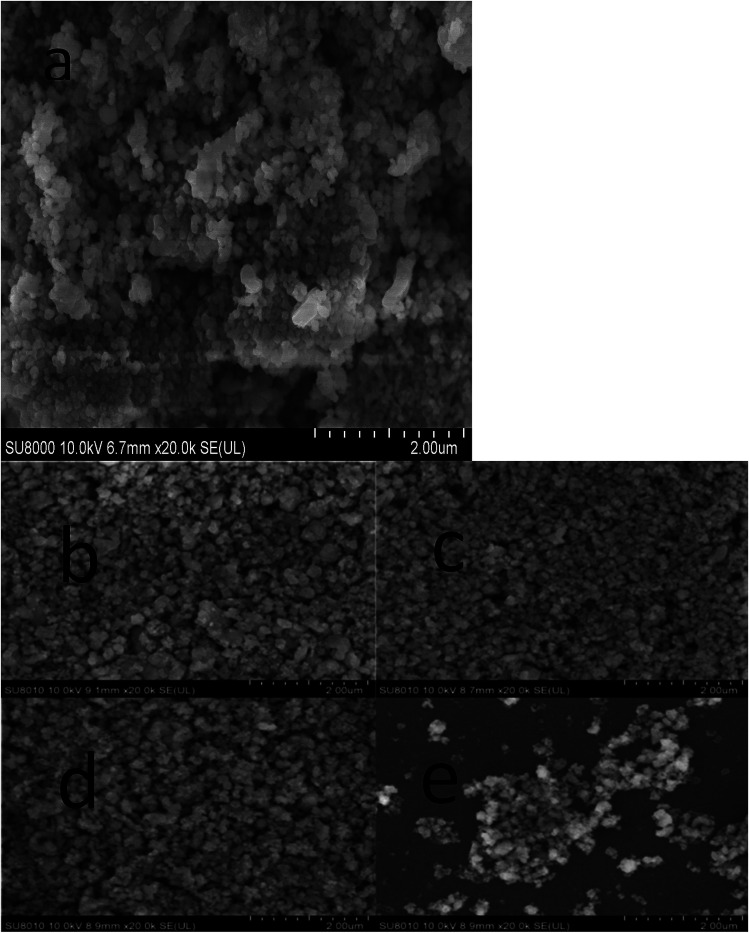
SEM photographs of ZnMn_2_O_4_ (a) and ZAMO0.5 (b), ZAMO1 (c), ZAMO2 (d), ZAMO10 (e).

**Fig. 4:**
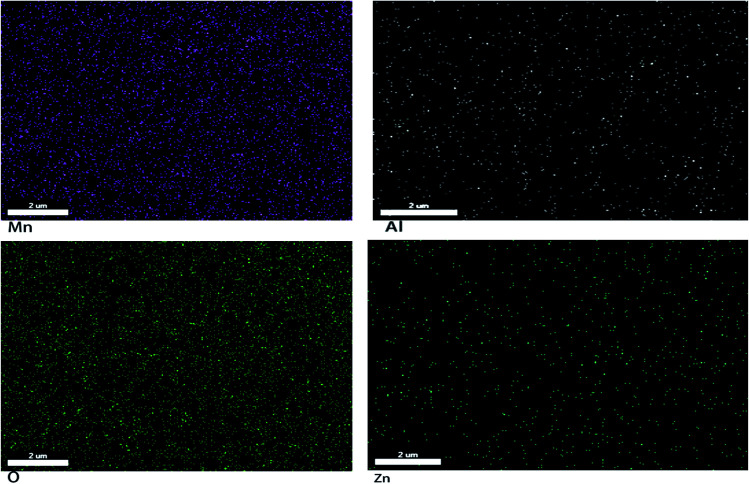
The EDX images of the ZAMO2.

Fig. 3 shows the SEM photographs of ZnMn_2_O_4_ and Zn_1−*x*_Al_*x*_Mn_2_O_4_ samples. All samples have an aggregated morphology of some irregular primary particles whose size is around 150 nm. There is no change in terms of the particle size and shape after the substitution of Al^3+^ for Zn^2+^. The EDX images of the ZAMO2 are shown in Fig. 4. It can be seen that the sample is composed of zinc, manganese, oxygen and aluminum and those elements are in uniform distribution. It further proves that the aluminum ions were successfully introduced into the ZnMn_2_O_4_ lattice.

**Fig. 5:**
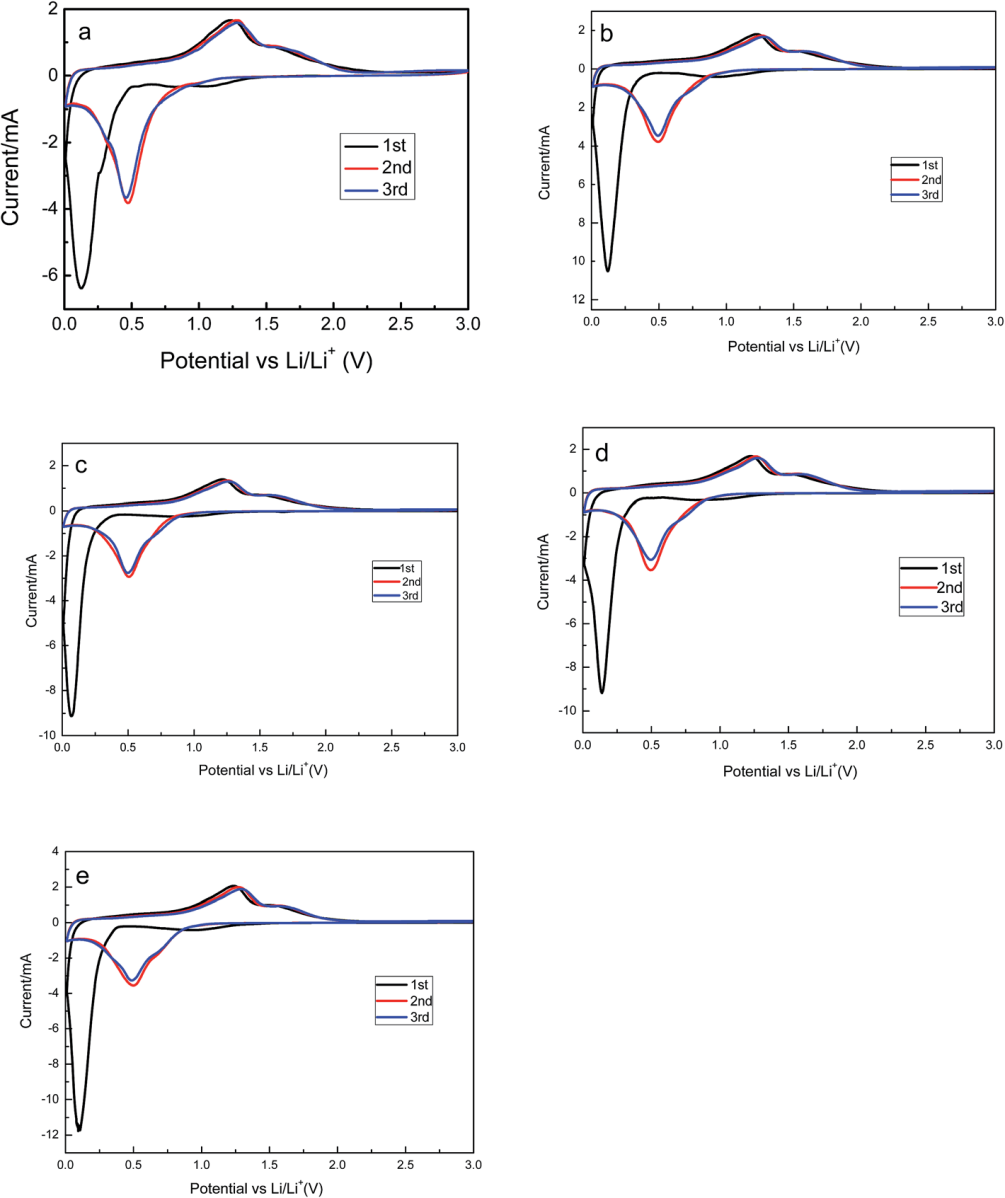
The cyclic voltammograms of ZnMn_2_O_4_ (a), ZAMO0.5 (b), ZAMO1 (c), ZAMO2 (d), ZAMO10 (e).

The cyclic voltammograms of Zn_1−*x*_Al_*x*_Mn_2_O_4_ electrode are provided in Fig. 5. All cells were operated at a scan of 0.1 mV s^−1^ in the voltage range of 0.01–3.0 V *versus* Li/Li^+^. In the case of the ZnMn_2_O_4_, there are three cathodic peaks and two anodic peaks in the first cycle. According to the discussion in literatures, the first peak located at 1.2 V is attributed to the reduction of Mn^3+^ to Mn^2+^.^36^ The second one located at 0.8 V is ascribed to the electrolyte decomposition along with the formation of solid-electrolyte interface (SEI) on the surface of the electrode. Both of those two peaks disappear in the following cycles. The sharp and intense peak at 0.13 V is attributed to reduction of Mn^2+^ and Zn^2+^ to metallic Mn and Zn nanoparticles, respectively.^37^ Simultaneously, the Li–Zn alloy is also formed at this low potential. There are two peaks in the first charge process, one is at 1.3 V, and the other is at 1.5 V, corresponding to the oxidation of metallic Mn and Zn nanoparticles to MnO and ZnO.^38^ In the second and the third cycles, there is only one peak located at 0.5 V in the discharge process. They are resulted from the reduction of MnO and ZnO to Mn and Zn. The similarity of the subsequent CV curves indicates the high electrochemical reversibility of the sample. The cyclic voltammograms of Zn_1−*x*_Al_*x*_Mn_2_O_4_ electrodes are shown in Fig. 5b–e. They are almost the same as the cyclic voltammograms of ZnMn_2_O_4_, implying that the Al^3+^ doping has no effect on the charge–discharge mechanism of ZnMn_2_O_4_.

**Fig. 6:**
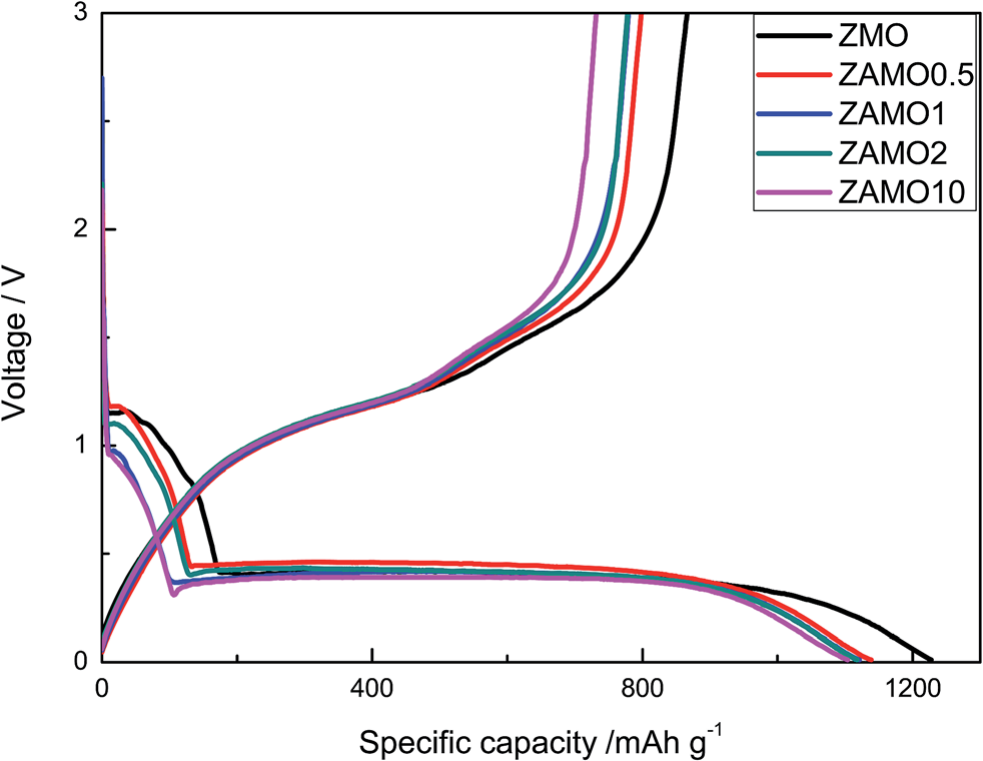
The first discharge–charge curves of ZnMn_2_O_4_ and Zn_1−*x*_Al_*x*_Mn_2_O_4_.

The first discharge–charge curves of ZnMn_2_O_4_ and Zn_1−*x*_Al_*x*_Mn_2_O_4_ are shown in Fig. 6. The discharge plateaus of ZnMn_2_O_4_ and Zn_1−*x*_Al_*x*_Mn_2_O_4_ are all at about 0.4 V. The specific capacities of the Zn_1−*x*_Al_*x*_Mn_2_O_4_ between 0.4 V and 1.2 V slightly decrease after the Al^3+^ doping. It is probably related to the decrease in Mn^3+^ concentration in Zn_1−*x*_Al_*x*_Mn_2_O_4_. Thus the total discharge capacities are decreased. The initial discharge capacities of five samples are much higher than the theoretical capacity of 1024 mA h g^−1^ corresponding to the reaction of ZnMn_2_O_4_ + 9Li^+^ + 9e^−^ → ZnLi + 2Mn + 4Li_2_O. The extra capacity could result from the SEI layer on the surface of the electrode.^39–42^ The coulombic efficiency (CE) is an important parameter of LIBs. It reflects the degree of the Li^+^ going back to the lattice. The charge retention capacity increases along with CE increasing. The CEs of ZnMn_2_O_4_ and Zn_1−*x*_Al_*x*_Mn_2_O_4_ are about 70% (1227/866, 1138/797, 1121/779, 1117/777, 1104/731 mA h g^−1^). This value is higher than CEs of other ZnMn_2_O_4_-based anodes reported in literature.^43–46^

**Fig. 7:**
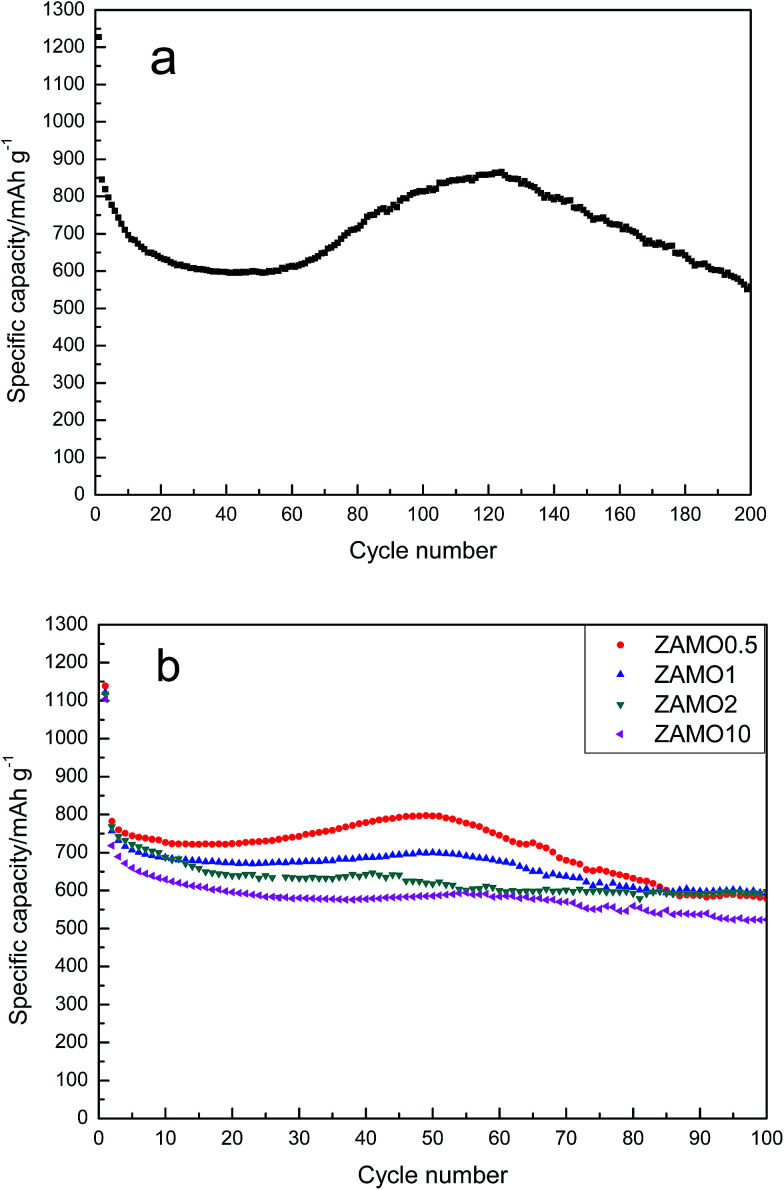
The discharge/charge curves of ZnMn_2_O_4_ (a) and Zn_1−*x*_Al_*x*_Mn_2_O_4_ (b).

The cyclic performances of ZnMn_2_O_4_ and Zn_1−*x*_Al_*x*_Mn_2_O_4_ are depicted in Fig. 7. All half-cells were operated at a current density of 100 mA h g^−1^ in the voltage range of 0.01–3.0 V in room temperature, using Li metal as an anode. The specific discharge capacity of ZnMn_2_O_4_ is gradually decreased till the 40th cycle and then increases till the 120th cycle. This abnormal increase in the discharge capacity between the 40th and 120th cycle have been ascribed to two factors. One is the formation of a polymer organic layer on the electrode during the cycling which can reserve Li reversibly.^47,48^ The other is the particles cracked during the cycling so that the contact area between the electrode and electrolyte solution increases.^49^ This phenomenon is not always good. When the nanostructure completely crumbled, the active materials will be separated from the conductive agent. This is bad for charge and ion transmission. As expected, following the rise, there is a quick decline in the capacity.

The discharge/charge curves of Zn_1−*x*_Al_*x*_Mn_2_O_4_ electrodes are shown in Fig. 7b. It is obvious that the fluctuation of capacity disappears gradually after the doping with Al and the cycleability tends to be steady. ZAMO2 has a reversible capacity of 597.7 mA h g^−1^.

**Fig. 8:**
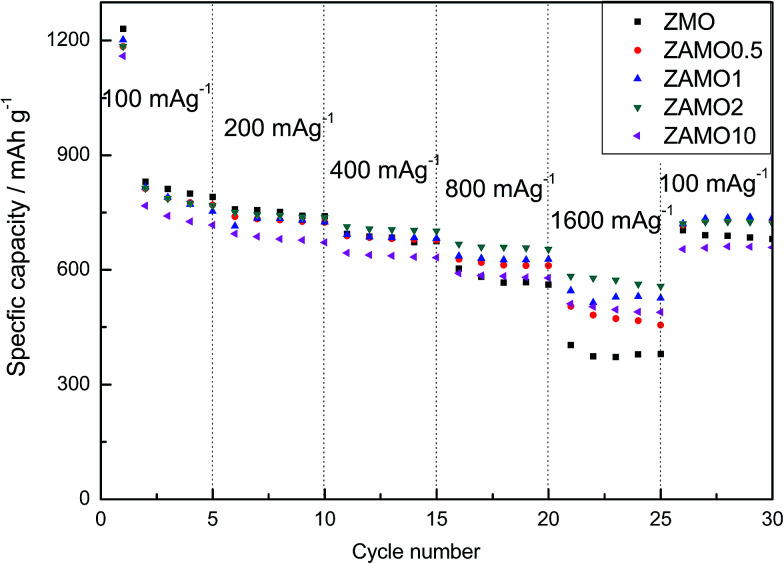
The rate capability of ZnMn_2_O_4_ and Zn_1−*x*_Al_*x*_Mn_2_O_4_.

The rate capabilities of five samples are shown in Fig. 8, in the picture, Zn_1−*x*_Al_*x*_Mn_2_O_4_ exhibits a much higher capacity at a current density of 1600 mA g^−1^. The capacities of ZAMO2 and ZMO are 557.8 mA h g^−1^ and 379.8 mA h g^−1^. When the current density goes back to 100 mA g^−1^, the capacity of ZAMO2 is also higher than ZMO. It indicates that ZAMO2 is more appropriate for charge/discharge at high current density.

**Fig. 9:**
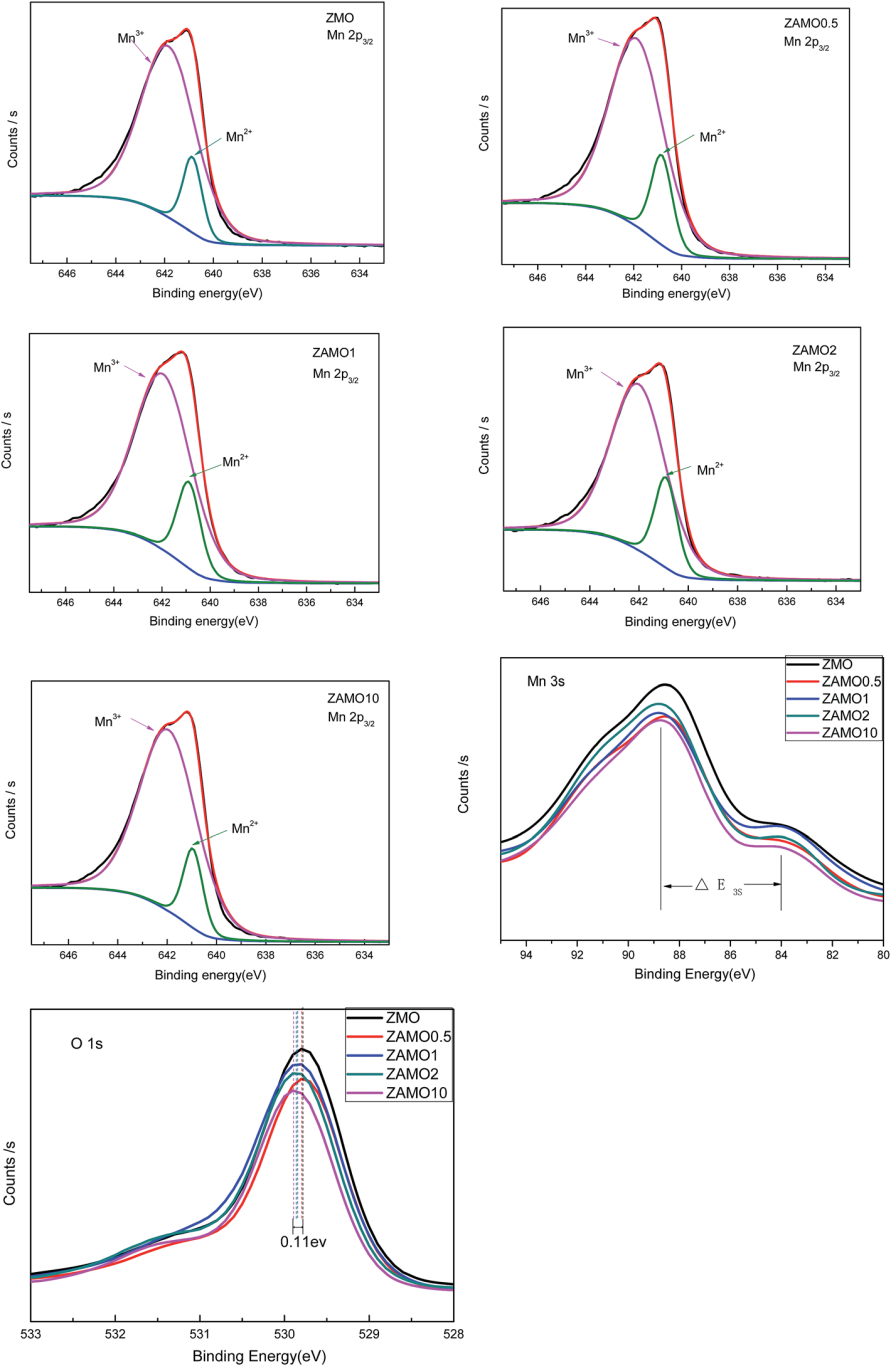
The X-ray Photoelectron Spectroscopy (XPS) of ZnMn_2_O_4_ and Zn_1−*x*_Al_*x*_Mn_2_O_4_.

**Fig. 10:**
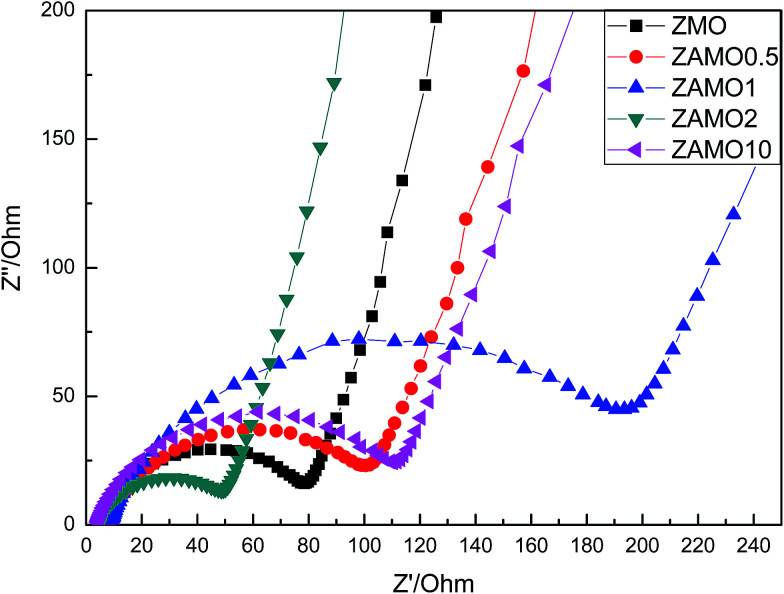
Nyquist plots of Zn_1−*x*_Al_*x*_Mn_2_O_4_ before cycling.

In order to clarify the mechanism of this improved performances after the introduction of Al, the X-ray Photoelectron Spectroscopy (XPS) and Electrochemical Impedance Spectroscopy (EIS) were measured and the results were provided in Fig. 9 and 10.

Fig. 9 shows the characteristic peaks of Mn_2p_, Mn_3s_ and O_1s_. The binding energy of Mn_2p_3/2__, multiplet splitting of the Mn_3s_ level, the binding energy of O_1s_ and the atomic ratio of Mn^3+^/Mn^2+^ are list in Table 1. The binding energy of Mn_2p_3/2__ for the samples of Zn_1−*x*_Al_*x*_Mn_2_O_4_ slightly shifts from 641.1 eV to 641.2 eV, while the binding energy of O_1s_ shifts from 529.8 eV to 529.9 eV, when *x* increases from 0 to 0.1. These results implied that the substitution of Al for Zn increases the interaction between Mn and O. As to the Δ*E*_3s_ of Mn, it increases from 4.5 eV to 5.1 eV when *x* is from 0 to 2%. According to previous research,^50^ the higher Δ*E*_3s_ value indicated a lower valence of Mn. It is well known that Zn in ZnMn_2_O_4_ exhibits +2 and Mn is +3, after the substitution of Al^3+^ for Zn^2+^, the valence of Mn should be lower so that the sample displays electric neutrality. Furthermore, the atomic ratio of Mn^3+^/Mn^2+^ calculated through XPS analysis decreases from 6.5 to 4.5 when *x* is from 0 to 2%. Those results prove that some Mn^3+^ in Zn_1−*x*_Al_*x*_Mn_2_O_4_ is partly transformed to Mn^2+^ in order to balance the Al^3+^ doping.

**Table 1:** Binding energy (eV) and the atomic ratio of Mn^3+^/Mn^2+^ of Zn_1−*x*_Al_*x*_Mn_2_O_4_

	Binding energy, Mn_2p_3/2__	Δ*E*_3s_	Binding energy, O_1s_	The atomic ratio of Mn^3+^/Mn^2+^
ZMO	641.1	4.5	529.8	6.5
ZAMO0.5	641.1	4.7	529.8	5.3
ZAMO1	641.2	4.9	529.8	4.9
ZAMO2	641.2	5.1	529.9	4.5
ZAMO10	641.2	4.5	529.9	6.6

Fig. 10 shows the Nyquist plots of Zn_1−*x*_Al_*x*_Mn_2_O_4_ before cycling. The depressed semicircles in the high frequency region are related to the ohmic resistance (*R*_e_) and the charge transfer resistance (*R*_ct_) through the electrode/electrolyte interface.^51–53^ Low resistance is good for the charge and ion diffusion. From the figure, we can easily find that ZAMO2 has the least diameter of the semicircles.

**Fig. 11:**
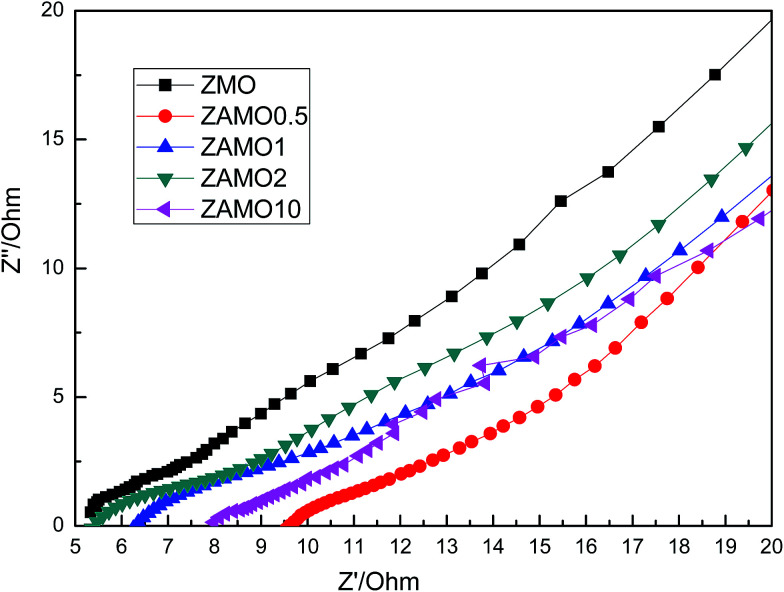
Nyquist plots of Zn_1−*x*_Al_*x*_Mn_2_O_4_ after 10 cycles.

Fig. 11 shows the Nyquist plots of Zn_1−*x*_Al_*x*_Mn_2_O_4_ after 10 cycles. The curves are almost lines with a slope of 45 degrees. This indicates that the batteries are controlled by the diffusion step of Li^+^ in solid phase.

According to the result of XPS and EIS, we believed that the introduction of Al makes Mn into a mixed valence state which can shorten the band gap width of manganese oxide and result in materials with higher conductivity. SEM shows that the particle size and morphology have no change after the doping, so that this improvement is all result from the charge compensation after the nonequivalent substitution.

## Conclusion

In summary, the nonequivalent substitution of ZnMn_2_O_4_ was successfully realized by spray drying process following with annealing treatment. Results of XRD, EDX and XPS indicate that the materials are pure and the aluminum ions are successfully introduced into the ZnMn_2_O_4_ lattice. Meanwhile, the valance of Mn in Zn_1−*x*_Al_*x*_Mn_2_O_4_ is in a mixed state because of charge compensation. ZAMO2 exhibits excellent stability, which maintains a high reversible of 597.7 mA h g^−1^ at the current density of 100 mA g^−1^ after 100 cycles. Even at high current density of 1600 mA g^−1^, the reversible capacity is still kept at 557.8 mA h g^−1^, which is higher than the capacity of theoretical commercial graphite. The electrochemical results demonstrated that the nonequivalent-substitution is a successful try for development of advanced anode material for high-performance LIBs.

## Conflicts of interest

There are no conflicts to declare.

## Supplementary Material
